# Direct Demonstration of Treponema Pallida in Brain of Paretic

**Published:** 1914-06

**Authors:** 


**Direct Demonstration of Treponema Pallida in Brain of Paretic.** Geo. W. Brock of Peoria, Ill..Med. Jour., May, 1914, reports a case, using the Giemsa stain and refers to other cases in the literature.

